# Resected primary mucinous cholangiocarcinoma of the liver

**DOI:** 10.1186/s40792-018-0450-3

**Published:** 2018-05-02

**Authors:** Kei Hagiwara, Kenichiro Araki, Takahiro Yamanaka, Norihiro Ishii, Takamichi Igarashi, Akira Watanabe, Norio Kubo, Norifumi Harimoto, Hiroyuki Kuwano, Sumito Nobusawa, Shinichi Aishima, Ken Shirabe

**Affiliations:** 1 Hepatobiliary and Pancreatic Surgery, Gunma University Graduate School of Medicine, 3-39-22 Showa-machi, Maebashi, 371-8511 Japan; 2 General Surgical Science, Gunma University Graduate School of Medicine, Maebashi, Gunma Japan; 3 Department of Human Pathology, Gunma University Graduate School of Medicine, Maebashi, Gunma Japan; 4 Pathology and Microbiology, Saga University, 5-1-1 Nabeshima, Saga, 849-8501 Japan

**Keywords:** Combined carcinoma, Intraductal papillary neoplasm of the bile duct, Metastatic liver tumors, Mucinous cholangiocarcinoma

## Abstract

**Background:**

Mucinous cholangiocarcinoma (MC) is a very rare variant of intrahepatic cholangiocarcinoma. MC is characterized by rapid growth, widespread metastasis, and poor prognosis. We report a case of resected MC of the liver.

**Case presentation:**

We found a 13.6-cm hypovascular tumor in the left hepatic lobe of a 68-year-old man, which we initially diagnosed as a mass-forming intrahepatic cholangiocarcinoma. Left lobe and caudate resection was performed without major intraoperative or postoperative complications. He was discharged home on postoperative day 9 and had no recurrence for 6 months. Pathological examination showed a mucous lobulated tumor with abundant mucus in the cytoplasm and extracellular regions. After differential diagnosis that considered invasive intraductal papillary neoplasm of the bile duct and metastatic liver tumors from the digestive tract, this tumor was diagnosed as a cholangiocarcinoma rare variant: primary mucinous carcinoma of the liver.

**Conclusion:**

Analysis of previous reports suggests that primary MC of the liver could be classified into two subtypes: pure MC and combined hepatocellular carcinoma and MC. Notably, the latter has been reported only in patients with chronic liver disease, whereas the former has only been reported in patients with no underlying disease.

## Background

Mucinous cholangiocarcinoma (MC) is a very rare variant of intrahepatic cholangiocarcinoma, which contains a large amount of mucin [1]. It must therefore be differentiated from invasive intraductal papillary neoplasm of the bile duct (IPNB) and metastatic liver tumors from the digestive tract. MC is characterized by rapid growth, widespread metastasis, and poor prognosis [2]. Here, we report a case of resected mucinous cholangiocarcinoma of the liver.

## Case presentation

**Fig. 1:**
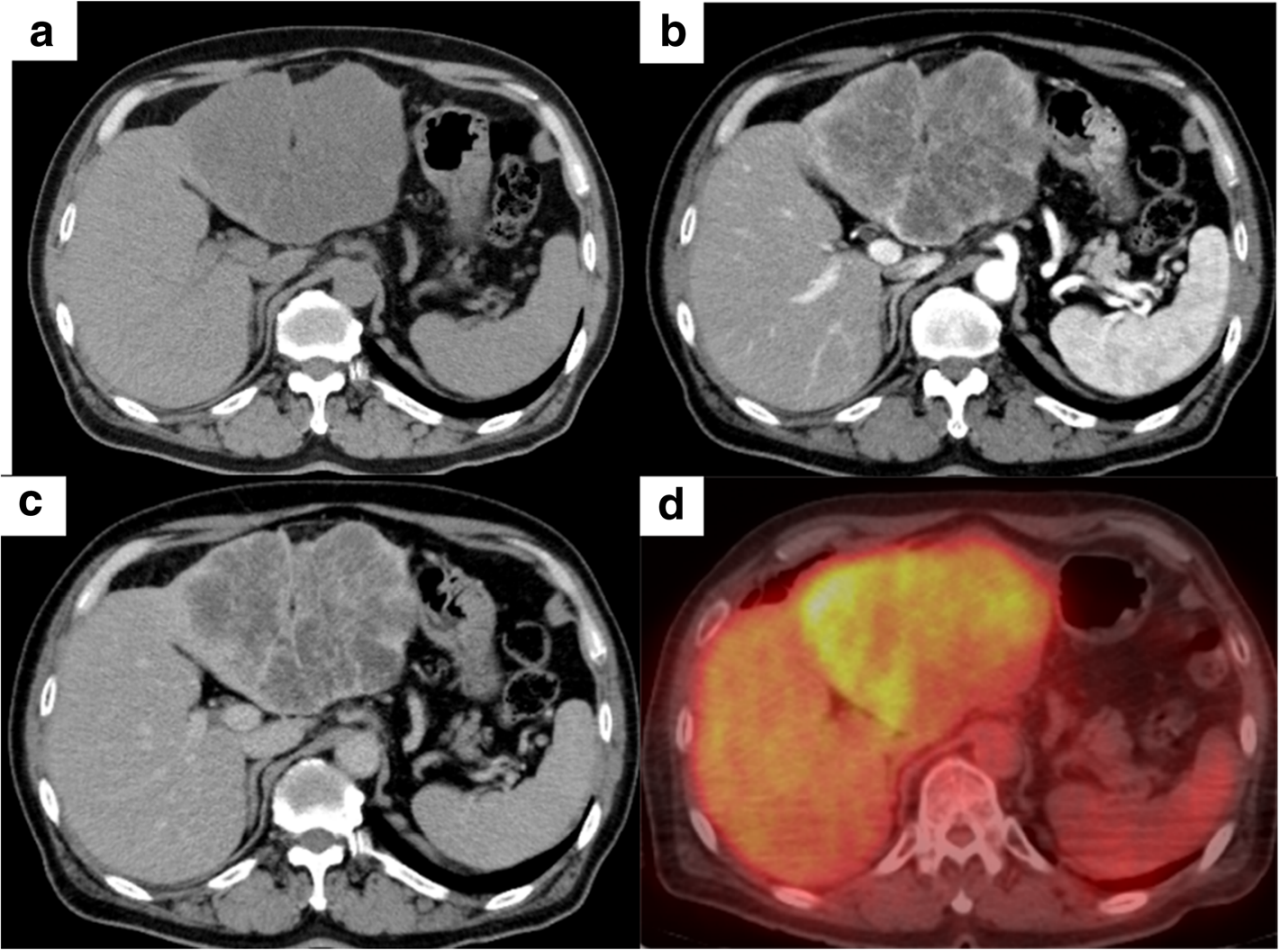
**a** Plain computed tomography (CT) scan showed a 13.6-cm low-density mass in left hepatic lobe. **b** Dynamic CT arterial phase scan shows enhanced edge of the mass with non-enhanced interior. **c** Low-density area remained in the equilibrium phase. **d** Fluorodeoxyglucose-positron emission tomography (FDG-PET) demonstrated a high FDG uptake (SUVmax 4.9) in the peripheral region of the mass

The patient, a 68-year-old man, was admitted with a complaint of epigastralgia and anorexia. Computed tomography (CT) showed a 13.6-cm hypovascular tumor in his left hepatic lobe, and he was referred to our hospital. No notable abnormalities were observed in his physical examination nor in his blood examination, including hepatobiliary enzymes. His tumor marker levels were carcinoembryonic antigen (CEA), 81.5 ng/ml; carbohydrate antigen (CA) 19-9, 28 U/ml; α-fetoprotein (AFP), 5.0 ng/ml; and protein induced by vitamin K absence or antagonists-II (PIVKA-II), 14 mAU/m. A CT scan showed a 13.6-cm hypovascular tumor in his left hepatic lobe (Figs. 1a–c). Fluorodeoxyglucose-positron emission tomography (FDG-PET) demonstrated high FDG uptake (SUVmax 4.9) in the peripheral region of the mass (Fig. 1d). Magnetic resonance imaging (MRI) showed a large, irregular lobulated mass (major diameter 128 mm) in the lateral segment of the liver, with low signal intensity on T1-weighted images and high intensity on T2-weighted images. We observed no change in his preoperative esophagogastroduodenoscopy or colonofiberscopy; no evidence of intrahepatic metastasis, distant metastasis, or lymph node metastasis on systemic CT; and no abnormal uptake on FDG-PET.

From this investigation, we diagnosed this tumor as intrahepatic cholangiocarcinoma, mass-forming type. We performed a left lobe and caudate resection without major intraoperative complications (surgical time 379 min; total bleeding 306 g; resected liver weight: 821 g). This patient had no postoperative complication and was discharged home on postoperative day 9. He underwent adjuvant chemotherapy (TS-1™) and had no recurrence for 6 months.

**Fig. 2:**
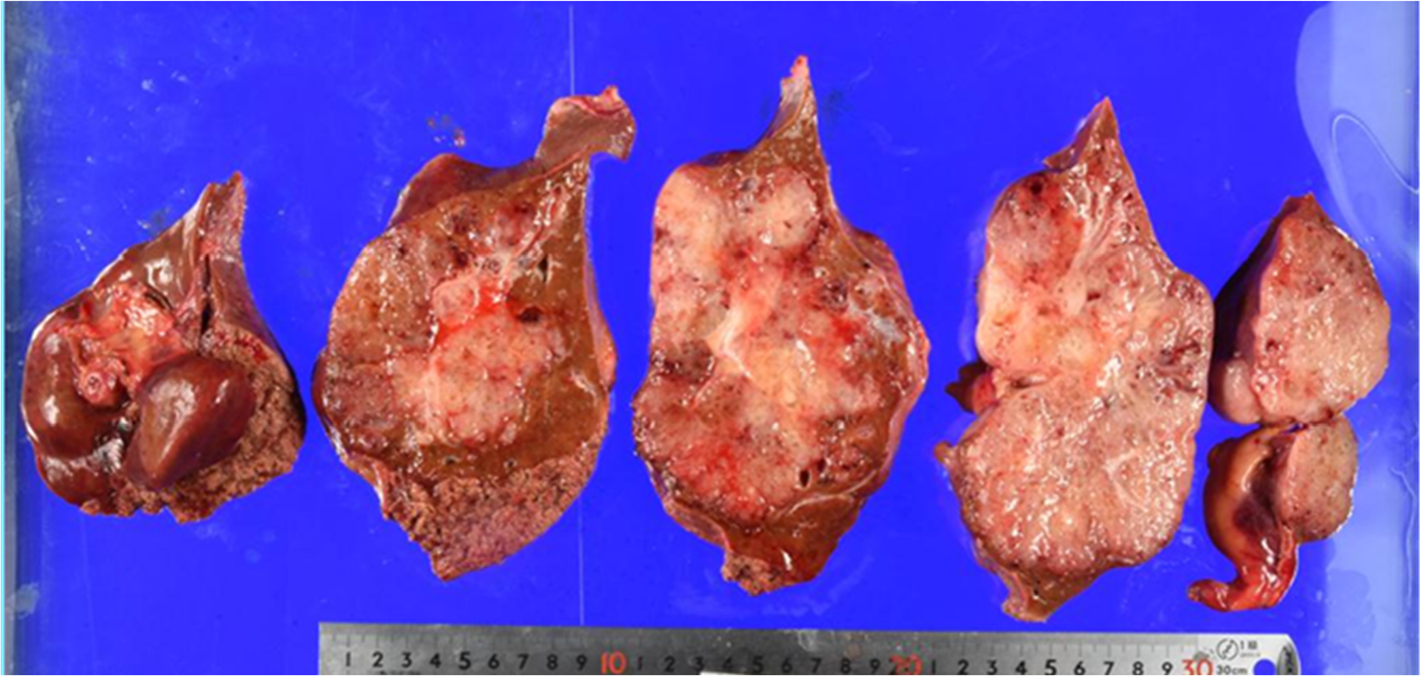
Resected liver specimen shows a mucous lobulated tumor that occupied most of the left lobe

**Fig. 3:**
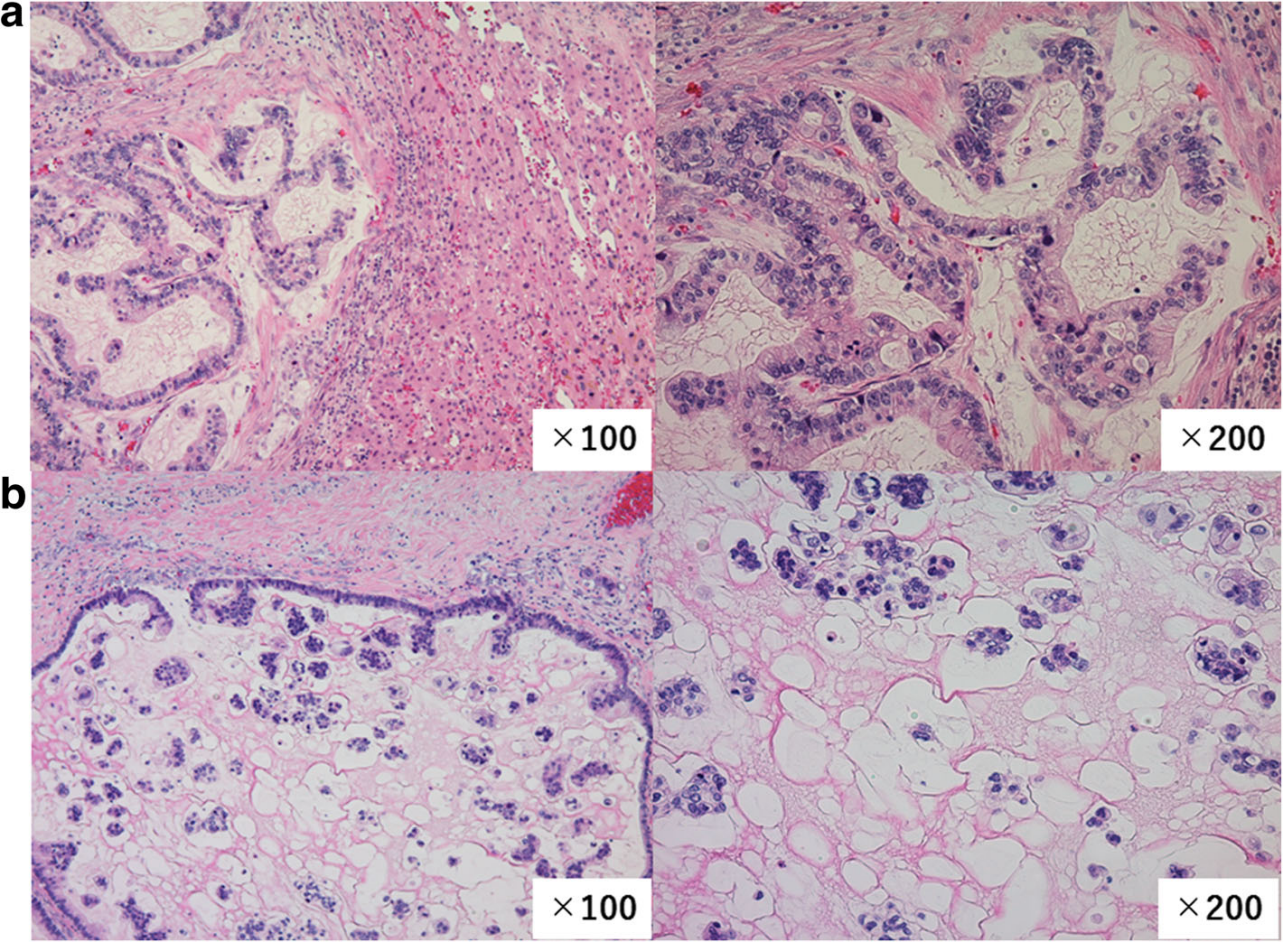
**a** Columnar tumor cells with abundant cytoplasmic mucus arranged in papillary or tubular structure (HE staining). Original magnification: × 200 (right) and × 100 (left). **b** Abundant mucus at extracellular region; tumor cells observed in mucus lakes (HE staining). Original magnification: × 200 (right) and × 100 (left)

**Fig. 4:**
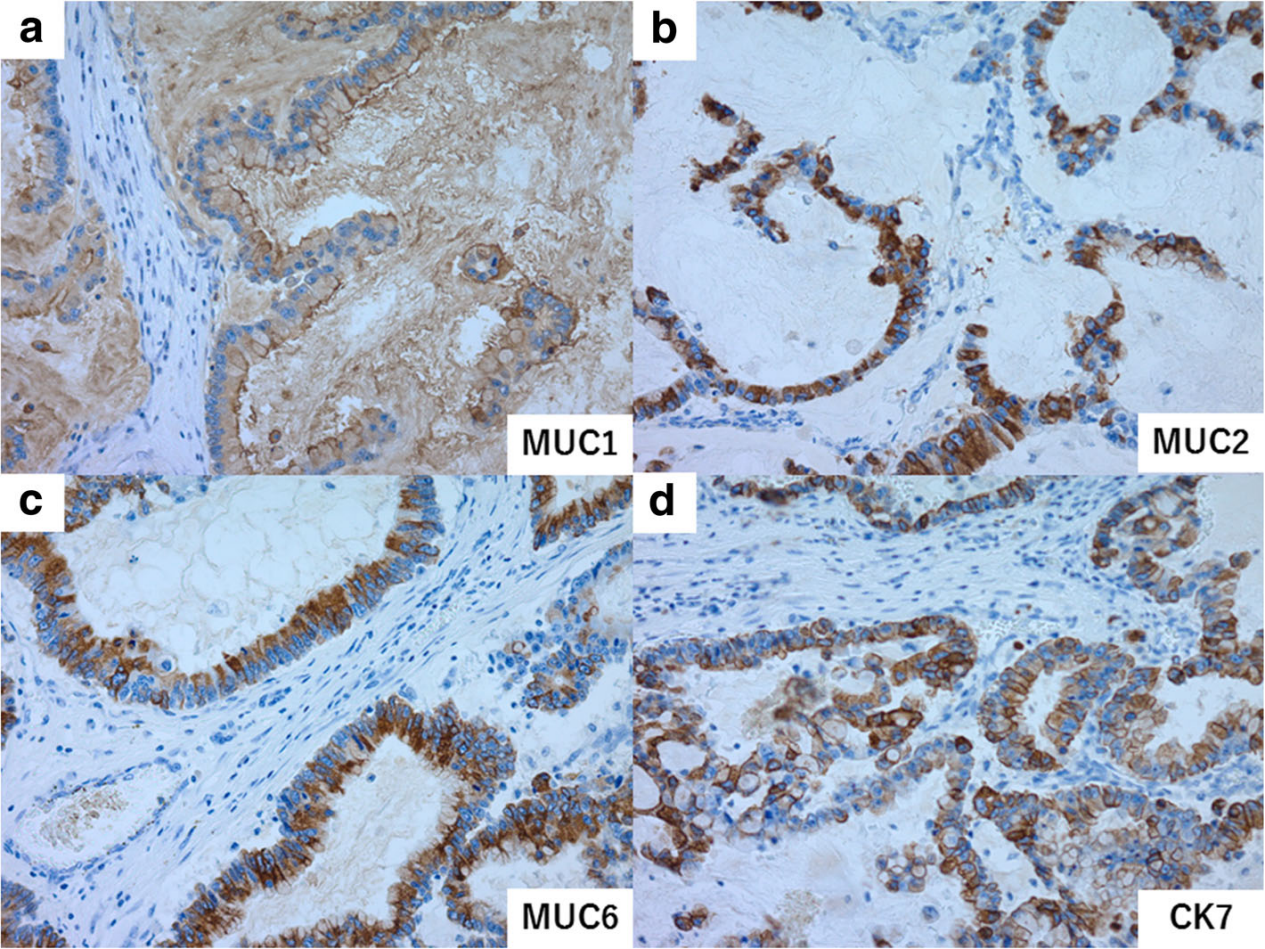
Positive immunohistochemical staining for **a** MUC1, **b** MUC2, **c** MUC6, and **d** CK7

Macroscopic pathological examination found a mucous lobulated tumor that occupied most of the resected left liver lobe (Fig. 2). In microscopic examination, columnar tumor cells with abundant cytoplasmic mucus were arranged as tubular or low-papillary structures without delicate fibrovascular cores (Fig. 3a). Profuse extracellular mucus was also observed in the region, with tumor cells in mucus lakes (Fig. 3b). Although small areas of non-invasive growth were seen to line the luminal bile duct surface, most of the tumor cells infiltrated the liver parenchyma. Cancer cells were microscopically seen to have invaded the portal vein, but the resection margin was negative. These tumor cells produced abundant mucus; histological staining was positive for MUC1, MUC2, MUC5AC, and MUC6. Staining for cholangiocarcinoma marker (CK7) was positive, but colorectal carcinoma marker (CK20 and CDX2) and HCC marker (Hep-Par1, AFP, and Glypican3) were negative (Figs. 4a–d). Therefore, we classified this tumor as a cholangiocarcinoma rare variant, mucinous carcinoma.

## Discussion

Mucinous cholangiocarcinoma is a very rare variant of intrahepatic cholangiocarcinoma. Only one case has been reported among 462 patients with cholangiocarcinoma investigated by Nakanuma et al. [1]. We found only 11 case reports of mucinous cholangiocarcinoma in the literature (summarized in Table 1) [2–12].

**Table 1:** Summary of clinicopathological features in reported cases of mucinous cholangiocarcinoma, including the current case

	Pure MC	Combined HCC and MC	*P* value
Number	8 cases (including this case)	4 cases	
Underlying liver disease	none of 8/8 cases	HCV 2 cases, alcoholic cirrhosis 1 case, cryptogenic cirrhosis 1 case	0.0009
Age (years)	41–79(average 65 years old)	51–70s (average 58 years old)	
Sex (male:female)	5:3	2:2	0.679
Tumor size (cm)	6.7 ± 3.7	7.6 ± 5.0	0.744
Tumor markers positivity			
CEA	4/6 (66%)	3/4 (75%)	0.778
AFP	0/6 (0%)	3/4 (75%)	0.0112

*AFP* α-fetoprotein, *CEA* carcinoembryonic antigen, *HCC* hepatocellular carcinoma, *HCV* hepatitis C, *MC* mucinous cholangiocarcinoma

MC contains a large amount of mucin and so requires differentiation from IPNB and metastatic liver tumors from the digestive tract, especially colorectal mucinous carcinoma. Invasive IPNB originates in the intrahepatic large bile ducts and shows a pattern of intraductal growth. Histologically, it is a well-differentiated papillary adenocarcinoma and contains a non-invasive dysplastic epithelium lesion [13]. This case showed a mass-forming growth pattern, with no discernible papillary histological constructions or cyst-like structures in offshoots of the intrahepatic large bile ducts. We also did not detect papillary projections with delicate fibrovascular cores in cystically dilated bile ducts. Therefore, we did not diagnose this case as invasive IPNB.

Our differential diagnosis also included liver metastasis from colorectal mucinous carcinoma. In this case, there are no changes in the preoperative esophagogastroduodenoscopy and colono fiberscope and no abnormal uptake of gastrointestinal tracts on FDG-PET. Immunohistochemistry revealed positive staining for cytokeratin 7 and negative staining for cytokeratin 20 and CDX 20. Chu et al. reported that more than 80% of colorectal cancer has CK7-/CK20+ profiles [14]. CDX2 was expressed in almost all colorectal cancers [15]. So, we denied the possibility of liver metastasis of colorectal cancer.

Of the 11 previously reported cases of primary MC of the liver, 4 were combined carcinomas with components of hepatocellular carcinoma (HCC) [3, 10–12]. All of these four cases suggest that HCC components and MC components in combined carcinomas might originate from the same cancer cells. In contrast, pure MC had no components of HCC, although pathological features of pure MC resembled those of MC components in the combined carcinomas [2, 4–9].

Each of the four patients with combined carcinomas had chronic liver diseases (two with hepatitis C, one with alcoholic cirrhosis, and one with cryptogenic cirrhosis), whereas none of eight pure MC cases (including the present case) had chronic liver disease—a significant difference in underlying liver diseases between the two groups (*P* = 0.0009).

With respect to tumor markers, AFP was elevated in three of the four combined carcinoma cases and none of MC cases (*P* = 0.0112); CEA was elevated in three of the four combined carcinoma cases and four of six MC cases (*P* = 0.7782). No significant difference was recognized for age, sex, or tumor size. If pure MC was caused by the replacement of all HCC components with MC components, prevalence of underlying liver diseases of these two tumors would be similar and pure MC tumors would be larger than combined carcinomas. However, the underlying liver diseases were completely different, and tumor sizes made no obvious difference. Therefore, we think that these two tumors may come from different cells.

Mucin-producing cells of bile ducts are located in peribiliary glands (PBGs). Mucous glands in PBGs can be divided into intramural type (simple tubular mucous glands) and extramural type (branched tubuloalveolar seromucous glands) [16]. Cardinale et al. reported biliary tree stem/progenitor cells (BTSCs) in PBGs, with the potential to differentiate into hepatocytes, cholangiocytes, and pancreatic cells [17]. Nakanuma reported that IPNB and intraductal papillary neoplasm of the pancreas share common histologic and phenotypic features and biological behaviors [16], which suggests that IPNB could arise from ectopic pancreatic tissues in PBGs. Similarly, the two types of MC (pure MC and combined MC) might occur in PBGs, with the pure type arising from mucin-producing cells and the combined type arising from BTSCs.

Nelson et al. reported hepatic stem cells called oval cells in rodents. Oval cells express markers of both fetal hepatocytes and biliary cells and are capable of generating hepatocytes and bile duct cells. Oval cells appear in some types of liver injury and perform a liver regeneration. Similarly, intra-hepatic progenitor cells appear after liver injury and perform a liver regeneration in human adults are reported. [18]. As described above, all of the four patients with combined MC had chronic liver diseases. So, there is the possibility that combined MC arise from these intra-hepatic progenitor cells.

We observed nothing in this case that suggested the presence of HCC: no HCC components in the tumor; negative immunostaining for Hep-Par1, glypican-3, and AFP; no chronic liver diseases; and normal serum AFP levels. We therefore diagnosed this case as pure MC.

## Conclusions

Primary MC of the liver can be classified into two subtypes: pure MC, which apparently arises from livers with no chronic disease and MC combined with HCC, which arises from livers with chronic disease. This case appeared to be pure MC.

## Abbreviations

AFP: α-Fetoprotein
BTSCs: Biliary tree stem/progenitor cells
CA19-9: Carbohydrate antigen 19-9
CEA: Carcinoembryonic antigen
CK: Cytokeratin
CT: Computed tomography
FDG-PET: Fluorodeoxyglucose-positron emission tomography
HCC: Hepatocellular carcinoma
Hep-Par1: Hepatocyte paraffin 1
IPNB: Intraductal papillary neoplasm of the bile duct
MC: Mucinous cholangiocarcinoma MRI Magnetic resonance imaging
MUC: Mucin
PIVKA-II: Vitamin K absence or antagonists-II
